# Increased survival of cirrhotic patients with septic shock

**DOI:** 10.1186/cc12687

**Published:** 2013-04-19

**Authors:** Bertrand Sauneuf, Benoit Champigneulle, Alexis Soummer, Nicolas Mongardon, Julien Charpentier, Alain Cariou, Jean-Daniel Chiche, Vincent Mallet, Jean-Paul Mira, Frédéric Pène

**Affiliations:** 1 Medical intensive care unit, Cochin Hospital, Hôpitaux Universitaires Paris Centre, Assistance Publique des Hôpitaux de Paris, 27 rue du Faubourg Saint-Jacques, 75014 Paris, France; 2 Université Paris Descartes, Sorbonne Paris Cité, Faculté de Médecine, 15 rue de l'école de Médecine, 75014 Paris, France; 3 INSERM U970, Paris Cardiovascular Research Center (PARCC), European Georges Pompidou Hospital, 56 rue Leblanc, 75015 Paris, France; 4 Institut Cochin, INSERM U1016, CNRS UMR-8104, 22 rue Méchain, 75014 Paris, France; 5 Hepatology unit, Cochin Hospital, Hôpitaux Universitaires Paris Centre, Assistance Publique des Hôpitaux de Paris, 27 rue du Faubourg Saint-Jacques, 75014 Paris, France

## Abstract

**Introduction:**

The overall outcome of septic shock has been recently improved. We sought to determine whether this survival gain extends to the high-risk subgroup of patients with cirrhosis.

**Methods:**

Cirrhotic patients with septic shock admitted to a medical intensive care unit (ICU) during two consecutive periods (1997-2004 and 2005-2010) were retrospectively studied.

**Results:**

Forty-seven and 42 cirrhotic patients presented with septic shock in 1997-2004 and 2005-2010, respectively. The recent period differed from the previous one by implementation of adjuvant treatments of septic shock including albumin infusion as fluid volume therapy, low-dose glucocorticoids, and intensive insulin therapy. ICU and hospital survival markedly improved over time (40% in 2005-2010 *vs*. 17% in 1997-2004, *P *= 0.02 and 29% in 2005-2010 *vs*. 6% in 1997-2004, *P *= 0.009, respectively). Furthermore, this survival gain in the latter period was sustained for 6 months (survival rate 24% in 2005-2010 *vs*. 6% in 1997-2004, *P *= 0.06). After adjustment with age, the liver disease stage (Child-Pugh score), and the critical illness severity score (SOFA score), ICU admission between 2005 and 2010 remained an independent favorable prognostic factor (odds ratio (OR) 0.09, 95% confidence interval (CI) 0.02-0.4, *P *= 0.004). The stage of the underlying liver disease was also independently associated with hospital mortality (Child-Pugh score: OR 1.42 per point, 95% CI 1.06-1.9, *P *= 0.018).

**Conclusions:**

In the light of advances in management of both cirrhosis and septic shock, survival of such patients substantially increased over recent years. The stage of the underlying liver disease and the related therapeutic options should be included in the decision-making process for ICU admission.

## Introduction

The overall incidence of severe sepsis and septic shock is steadily increasing due to the aging of the population and to the growing prevalence of underlying co-morbidities including chronic organ dysfunctions and immunosuppression [1,2]. Several studies have highlighted the major influence of cirrhosis on the susceptibility to severe bacterial infections [3,4]. Indeed, the overall mortality rate of septic shock remains particularly high in cirrhotic patients, ranging from 60% to 100% [5-7], raising the question of indications of aggressive and extensive organ failure support in such patients.

The overall outcome of septic shock has clearly improved over the recent years, related to improved supportive care and rapid and protocolized treatment interventions supported by international guidelines [8]. Of note, the most significant improvements in survival from septic shock have been achieved in vulnerable subgroups including elderly patients, those with malignancies [9,10] or neutropenia [11]. Cirrhotic patients are usually excluded from interventional trials in sepsis, but it is likely that such a high-mortality subgroup would particularly benefit from therapeutic advances in septic shock. In addition, great strides have also been recently achieved in the management of specific complications of cirrhosis. Whether the survival gain achieved by therapeutic advances in septic shock also extends to cirrhotic patients has not been assessed. In order to determine the trend in mortality of cirrhotic patients with septic shock and the impact of related therapeutic interventions, we performed a retrospective single-center study over a 14-year time period.

## Materials and methods

### Patients and setting

The study took place in a 24-bed medical ICU with an average of 1,500 admissions per year. All patients with histological or clinical diagnosis of cirrhosis and presenting with septic shock at the time of ICU admission or within the first 48 h in the ICU were included. Septic shock was defined as a microbiologically proven or clinically suspected infection, associated with acute circulatory failure requiring vasoactive support despite adequate fluid filling [12]. Senior staffing remained quite stable over the 14-year study period. ICU admission decisions were taken on by both the intensivist and the referring hepatologist throughout the study period. Therefore, only patients with end-stage liver disease declined for liver transplantation were not admitted to the ICU. End-of-life decisions to withhold or withdraw life support were taken on collectively when all participants were convinced that maintenance or increase of life-sustaining therapies was futile and that death would irremediably occur in a short-term manner.

Informed consent was waived since the study was retrospective and observational, in accordance with French regulation of clinical research. This epidemiologic study did not require ethical approval, in accordance with the standards of our local institutional review board.

### Intended care for cirrhotic patients with septic shock

Systematic screening for infection included clinical features (temperature, signs of shock), biological parameters (leukocytes), chest X-rays, and cultures of blood, sputum, urine, and ascites. As soon as infection was recognized, patients were promptly treated with empirical broad-spectrum antibiotic combination, depending on the site of infection, known colonization and previous antibiotic treatment. Antifungal therapy was added if fungal infection was suspected or documented. Antimicrobial treatment was narrowed after identification of the responsible pathogen. In addition, source control measures, such as surgery or removal of infected catheters were applied when necessary. Hemodynamic management included fluid resuscitation combined with continuous infusion of vasoactive drugs (mostly norepinephrin, while associated cardiac dysfunction prompted the use of either a combination of norepinephrin and dobutamine or epinephrin). Terlipressin was not used in combination with other vasoactive drugs. Endotracheal intubation and mechanical ventilation were performed in case of respiratory failure or coma. Renal replacement therapy (RRT), through either intermittent hemodialysis or continuous venovenous hemofiltration was initiated in case of acute renal failure, severe metabolic acidosis, or other life-threatening metabolic disorders.

Several adjuvant therapies were progressively implemented in septic patients over the study period. Thus, patients with acute respiratory distress syndrome (ARDS) were mechanically ventilated using a protective strategy with low tidal volume of 6 mL/kg of predicted body weight [13]. More generally, the plateau pressure was limited to 30 cm H_2_O in all ventilated patients. Intensive insulin therapy was used to maintain blood glucose between 4.4 and 8.1 mmol/L [14]. Low-dose corticosteroids (200 mg hydrocortisone per day) were administrated in vasopressor-dependent septic shock for 5 to 7 days [15]. Patients with at least two organ dysfunctions were considered for treatment with activated protein C in the absence of contraindication [16]. In order to assess whether these therapeutic advances resulted in improved survival in cirrhotic septic patients, we divided the whole cohort in two near-sized period groups, 1997-2004 (first period) and 2005-2010 (second period), in between the first guidelines of the Surviving Sepsis Campaign were published.

### Data collection

The following data were collected: demographic characteristics; Charlson co-morbidity index (excluding points for liver disease) [17]; functional status prior to ICU admission as assessed by the Knaus scale (A, prior good health, no functional limitation; B, mild to moderate limitation of activity because of a chronic disease; C, serious but not incapacitating restriction of activity; D, severe restriction of activity, including bedridden or institutionalized persons) [18]; stage of cirrhosis graded using the Child-Pugh classification [19]; and the Model for End-stage Liver Disease (MELD) score [20]. Child-Pugh score was computed prior to the current acute complication whereas MELD score was computed on the first day in the ICU. We also collected the infection characteristics including microbiological and clinical documentation and adequacy of initial antibiotic regimen within the early 48 h following the onset of infection, the organ failures supports including type and volume of fluid loading, mechanical ventilation and RRT, as well as adjuvant treatments of sepsis (intensive insulin therapy, low-dose glucocorticoids and activated protein C). The Simplified Acute Physiology Score 2 (SAPS II) and the Sequential Organ Failure Assessment (SOFA) score were calculated on the first day in the ICU [21,22]. Outcomes were in-ICU, in-hospital, and 6-month survival rates. Vital status was assessed using the medical records or the administrative hospital database. The outcome of patients followed up in another hospital or discharged home was requested to their referring hepatologist or their general practitioner.

### Statistical analysis

Results are reported as median (25th-75th percentile) or number (%) as appropriate. Categorical variables were compared with χ^2 ^or Fisher exact tests, and continuous variables were compared with the Mann-Whitney U test. Survival curves were obtained using the Kaplan-Meier method and compared using the log-rank test. To identify characteristics associated with hospital mortality, we used a logistic regression model. Variables that reached a *P *value < 0.1 were entered into a multivariate analysis. Inclusion of severity scores in the analysis precluded the inclusion of related variables in order to avoid colinearity. The goodness-of-fit of the model was evaluated by the Hosmer-Lemeshow statistic. Odds ratios (OR) and their 95% confidence intervals (CI) were computed. All test were two-sided and *P *values < 0.05 were considered statistically significant. All calculations were performed with SPSS software version 16 (SPSS, Chicago, IL, USA).

## Results

### Patients characteristics

**Figure 1:**
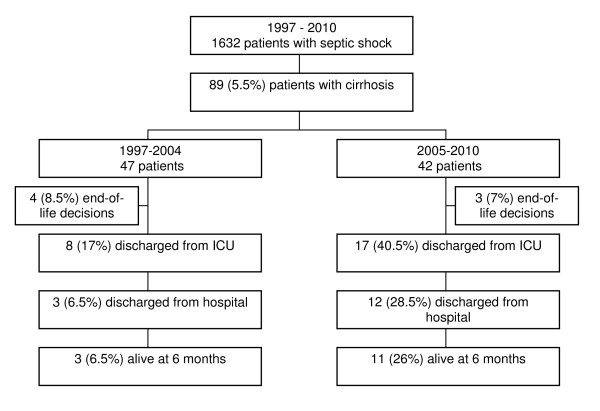
**Flow chart of the study**.

During the 14-year study period, 1,632 patients with septic shock were admitted to the ICU, including 89 patients (5.5%) with cirrhosis. Among them, 47 patients were admitted to the ICU during the first period (1997-2004) whereas 42 were admitted during the latter (2005-2010) (Figure 1). Underlying characteristics of patients are presented in Table 1 and were grossly similar between both study periods. Most cirrhosis was caused by chronic alcohol abuse associated or not with chronic viral hepatitis B or C infection. None of the patients presented with acute alcoholic hepatitis. Six patients had hepatocellular carcinoma, at an early stage (stage A, *n *= 2) or at an intermediate stage (stage B, *n *= 4), according to the BCLC classification [23].

**Table 1:** Baseline characteristics of patients.

Characteristic	1997-2004 47 patients	2005-2010 42 patients	*P*
Age (years, IQR)	55 (46.5-62)	58 (53-65)	0.08

Male gender	36 (76.5)	26 (62)	0.49

Knaus scale			0.15
Mild limitation (B)	6 (12.8)	10 (23.8)	
Important limitation (C)	25 (53.2)	17 (40.5)	
Severe limitation (D)	16 (34)	15 (35.7)	

Charlson score (IQR)^a^	1 (0-1)	1 (0-2)	0.51

Co-morbidities			
Chronic heart failure	4 (8.5)	3 (7)	0.81
COPD	2 (4.3)	3 (7)	0.79
Diabetes	6 (12.8)	10 (24)	0.27
Cancer	8 (17)	8 (19)	0.80
Hepatocellular carcinoma	3	3	
Other^b^	5	5	
Immunosuppression^c^	6 (12.8)	2 (4.8)	0.27

Cause of cirrhosis^d^			
Alcohol +/- virus	38 (80.1)	34 (80.1)	1
Chronic Hepatitis B virus infection	2 (4.3 )	0	0.50
Chronic Hepatitis C virus infection	6 (12.8)	4 (9.5)	0.74
Primary biliary cirrhosis	2 (4.3)	2 (4.8)	1
Undetermined	3 (6.4)	2 (4.8)	1

Persistent alcohol abuse	26 (55.3)	22 (52.3)	0.83

Nosocomial septic shock	13 (27.7)	14 (33.3)	0.67

Primary source of infection			
Respiratory	18 (38.3)	19 (45.2)	0.53
Abdominal	10 (21.3)	16 (38,1)	0.10
Spontaneous peritonitis	8 (17)	14 (33.3)	
Secondary peritonitis	2 (4.3)	2 (4.8)	
Urinary tract	6 (12.8)	4 (9.5)	0.49
Others			
CNS	3 (6.4)	0	0.10
Arthritis	1 (2.1)	1(2.4)	1
Isolated bacteriemia	2 (4.2)	0	0.50
Unknown	7 (14.9)	2 (4.8)	0.16

Bacteremia	20 (42.6)	14 (33.3)	0.39

Type of organisms			
Gram-positive cocci	15 (31.9)	13 (30.9)	1
Gram-negative bacilli	17 (36.2)	11 (23.8)	0.25
Fungi	2 (4.3)	2 (4.8)	1
Culture negative	12 (25.5)	8 (19)	0.61
Polymicrobial sepsis	1 (2.1)	8 (19)	0.01
Multi-drug resistant bacteria	2 (4.3)	8 (17)	0.04

Variables are expressed as number (percentage) or median (interquartile range (IQR)). ^a^Excluding liver disease points.

^b^Breast cancer (*n *= 3), colon cancer (*n *= 2), laryngeal cancer (*n *= 2), cholangiocarcinoma (*n *= 1), bladder cancer (*n *= 1), myeloma (*n *= 1).

^c^Infection by human immunodeficiency virus (*n *= 2), multiple myeloma (*n *= 1), hypogammaglobulinemia (*n *= 1), treatment with mesalazine (*n *= 1) and recent chemotherapy for cancer (*n *= 3).

^d^Sum of causes may exceed the number of patients because of concomitant alcoholic and viral cirrhosis.

CNS, Central Nervous System; COPD, Chronic Pulmonary Obstructive Disease.

The main sites of infections were pneumonia (42%), spontaneous or secondary peritonitis (29%), and urinary tract infection (11%). Sixty-nine patients (78%) had microbiologically documented infections balanced between gram-positive cocci (31%) and gram-negative bacilli (31%) (Table 1). Multi-drug resistant bacteria were more frequently involved in the recent period (Table 1).

### Management of organ failures

Major differences were noted in the management of septic shock between both study periods, including type and volumes of fluid loading within the first 3 days, ventilatory management, and adjuvant therapies of sepsis (Table 2). Indeed, intravenous albumin was frequently used in the most recent period (57.1% of patients *vs*. 8.5%, *P *< 0.001) whereas infusion of crystalloids was markedly reduced in the same time (3 (1.7-4.5) L *vs*. 6 (3-8.9) L, *P *< 0.001). Moreover, albumin-resuscitated patients tended to receive a higher albumin dose during the recent period (50 (30-72.5) *vs*. 20 (17.5-30) g, *P *= 0.06). RRT was less frequently required in the recent period (52.4 *vs*. 72.3%, *P *= 0.08). The ventilatory management also significantly differed between the two periods with smaller tidal volumes used in the period 2005-2010 (8.6 *vs*. 7 mL/kg, *P *= 0.001). Intensive insulin therapy and low-dose glucocorticoids were also more frequently used in the second period (83.3% *vs*. 31.9%, *P *< 0.001 and 81% *vs*. 44.7, *P *< 0.001, respectively). Only two patients were treated with drotrecogin alpha (activated) in the recent period.

**Table 2:** Organ failures, ICU management, and outcome.

Characteristic	1997-2004 47 patients	2005-2010 42 patients	*P*
Scoring systems (IQR)			
Child-Pugh score	9 (7-11)	10 (8.25-11)	0.22
MELD day 1	25 (17-33.8)	26 (20.2-32.8)	0.43
SAPS II day 1	56 (38-70.5)	59 (42-76)	0.10
SOFA day 1	14 (8.5-17.5)	13 (9-15)	0.18

Biological findings at ICU admission (IQR)			
Serum creatinine level, μmol/L	140 (96-199)	147 (105-235)	0.93
Serum bilirubin level, μmol/L	48 (35-110)	68 (44.2-140.5)	0.23
Arterial blood lactate level, mmol/L	4.2 (2-6.5)	4.3 (2.2-7.9)	0.68
Serum sodium, mmol/L	134 (130.5-139)	135.5 (129.2-139)	0.74
Serum protein level, g/L	54 (44.5-61)	59 (50-66.8)	0.05
Factor V, %	42 (33-64)	46 (33-60)	0.70
INR	2 (1.5-2.8)	2 (1.6-3.1)	0.69
White blood cells, 10^3^/mm^3^	13.6 (6.2-19)	10.7 (6.4-15.2)	0.45
C-reactive protein, mg/L	101 (60-167)	75 (34-127)	0.24

Mechanical ventilation	45 (95.7)	39 (93)	0.34
Tidal volume, mL/kg (IQR)^a^	8.6 (7.3-9.9)	7 (6.1-7.9)	<0.001
Lowest Pao_2_/Fio_2 _ratio (IQR)	101 (80-150)	117 (86-206)	0.17
ARDS	16 (34)	14 (33.3)	1
Renal replacement therapy	34 (72.3)	22 (52.4)	0.08

Antimicrobial treatment			
β-lactam	45 (95.7)	41 (97.6)	1
Quinolone	19 (40.4)	12 (25.5)	0.27
Aminoglycoside	23 (49)	22 (52.4)	0.83
Glycopeptide	9 (19.1)	6 (14.3)	0.58
Combination therapy	31 (64)	34 (81)	0.15

Inadequacy of initial antimicrobial treatment	5 (10.6)	3 (7.1)	1

Fluid loading within the first 3 days			
Crystalloids, L (IQR)	6 (3-8.9)	3 (1.7-4.5)	<0.001
Albumin resuscitation	4 (8.5)	24 (57.1)	<0.001

Adjuvant therapies of sepsis			
Intensive insulin therapy	15 (31.9)	35 (83.3)	<0.001
Low-dose glucocorticoids	21 (44.7)	34 (81)	<0.001
Activated protein C	0	2 (4.8)	1

ICU survival	8 (17)	17 (40)	0.02
Hospital survival	3 (6)	12 (29)	0.009
6-month survival	3 (6)	9 (21)	0.06

Variables are expressed as number (percentage) or median (interquartile range (IQR)).

^a^The predicted body weight was calculated using the following formulas: 50+0.91 (centimeters of height-152.4) (male patients) or 45.5+0.91(centimeters of height-152.4) (female patients).

ARDS, Acute Respiratory Distress Syndrome; INR, International Normalized Ratio; MELD, Model for End Stage Liver Disease; SAPS, Simplified Acute Physiology Score; SOFA, Sequential Organ Failure Assessment.

### Short-term and long-term outcomes

**Figure 2:**
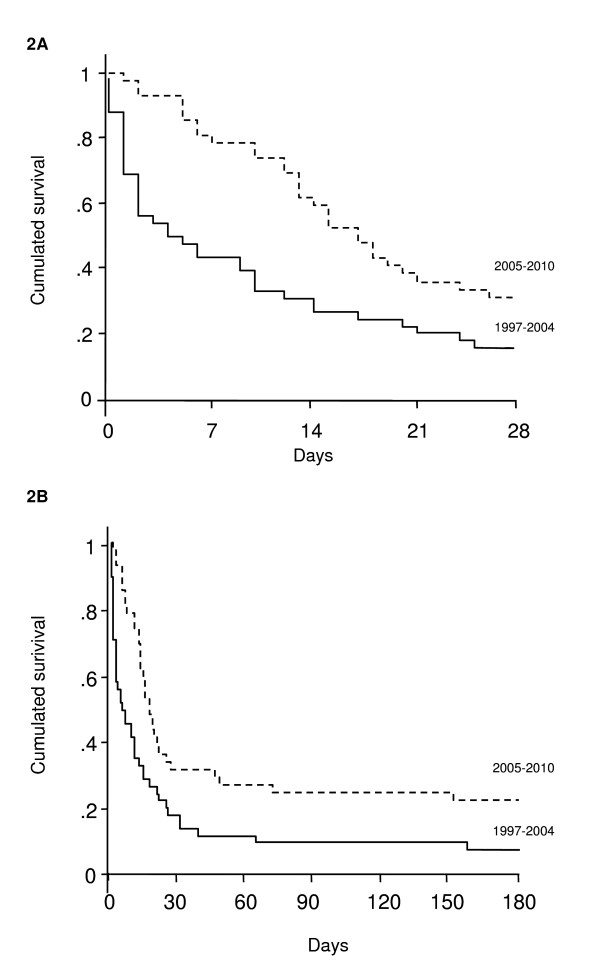
**Kaplan Meier estimates of 28-day (A) and 6-month (B) survival according to the period of admission (1997-2004, continuous line; 2005-2010, dotted line)**. Log-rank test: *P *= 0.02.

The rate of end-of-life decisions was similar between the two periods (Figure 1). The 6-month vital status was obtained for all patients. We observed a marked improvement in ICU and hospital survival rates in the recent period as compared to the 1997-2004 period (40% *vs*. 17%, *P *= 0.02 and 29% *vs*. 6%, *P *= 0.009, respectively). Most importantly, differences in survival occurred early in the course of the disease (Figure 2A). The benefit in short-term survival was sustained throughout the first 6 months following ICU admission (6-month survival rate 21% *vs*. 6%, *P *= 0.06) (Figure 2B). Two survivors of the recent period underwent liver transplantation within 6 months after ICU admission.

### Prognostic factors of hospital mortality

In order to identify the determinants of outcome, we compared the characteristics and treatments of hospital survivors and non-survivors (Table 3). Determinants of hospital mortality were the stage of the liver disease (Child-Pugh score, serum protein, and factor V levels), the extent of organ failures (day-1 SAPS II and SOFA scores, admission serum lactate level, renal replacement therapy), and admission during the first period. Finally, we carried out a multivariate logistic regression analysis taking into account the underlying liver disease stage (Child-Pugh score), the critical illness severity score (for example, SOFA score), and the recent changes in sepsis management (ICU admission period). When adjusted for admission SOFA score and age, the 2005-2010 period remained protective (OR 0.09, 95% CI 0.02-0.4) whereas the stage of cirrhosis prior to the acute complication was independently associated with hospital mortality (Child-Pugh score: OR 1.42 per point, 95% CI 1.06-1.9) (Table 4). Similar results were obtained when adjusted for SAPS II.

**Table 3:** Determinants of hospital outcome (univariate analysis).

Variable	Deceased 74 patients	Survivors 15 patients	*P*
Age (IQR)	56 (48-65)	58 (54-62.5)	0.6

Scoring systems (IQR)			
Child-Pugh score	10 (8-11)	7 (7-10)	0.05
MELD day 1	25 (18-33.5)	26 (18.5-33.5)	0.19
SAPS II day 1	59 (41.5-83)	50 (42-62.5)	0.02
SOFA day 1	14 (10-17)	9 (7.5-9)	0.03

Biological findings at ICU admission (IQR)			
Arterial blood lactate level, mmol/l	4.2 (2.1-7.3)	2.8 (2-4.5)	0.08
Serum protein level, g/L	54 (45-62)	62.5 (56-66)	0.06
Factor V, %	41 (27.5-62.5)	53 (48.5-70.5)	0.02

Renal replacement therapy	40 (65%)	5 (36%)	0.02
ARDS	22 (35.5%)	2 (13.5%)	0.08

Albumin resuscitation	24 (32.4)	4 (26.7)	0.77
Low-dose glucocorticoids	44 (59.5 )	11 (73.3)	0.39
Intensive insulin therapy	39 (52.7)	11 (73.3)	0.16

Admission period			
1997-2004	44 (59.5)	3 (20)	0.009
2005-2010	30 (40.5)	12 (80)	

Variables are expressed as number (percentage) or median (interquartile range (IQR)).

ARDS, acute respiratory distress syndrome; MELD, Model For End Stage Liver Disease; SAPS, Simplified Acute Physiology Score; SOFA, Sequential Organ Failure Assessment.

**Table 4:** Multivariate analysis of factors associated with in-hospital mortality.

Variable	Odds ratio	95% CI	*P*
SOFA (per point)	1.15	0.99-1.32	0.06
Age	1.02	0.96-1.08	0.53
Child-Pugh score (per point)	1.42	1.06-1.9	0.018
Period 2005-2010 (compared to 1997-2004)	0.09	0.02-0.4	0.004

CI, confidence interval, Goodness of fit (Hosmer-Lemeshow) chi-square *P *value = 0.54.

## Discussion

Septic shock represents a severe complication of cirrhosis with very low survival rates that question the relevance of life-sustaining therapies in this subgroup of patients. We report here that the current survival rate remains low but substantially improved over time, suggesting that advances in care of septic shock extended to this high-risk subgroup of patients. Most importantly, the short-term improvement in survival was sustained for at least 6 months, suggesting that ICU admission and extensive life support is justified in some patients. In addition to organ failures, the stage of liver disease as assessed by the Child-Pugh score appears to be an independent prognostic factor that should be taken into account in the decision-making process.

Cirrhosis is clearly associated with an increased predisposition to sepsis [4] and has been identified as an independent poor prognostic factor in patients with severe sepsis [24]. In addition, chronic alcohol abuse by itself may contribute to worsening organ failures and has been shown to be an independent risk factor of septic shock [25]. Multiple mechanisms concur to confer an increased susceptibility to bacterial infections and subsequently to multiple organ failure in cirrhotic patients. Bacterial translocation increases with the severity of liver disease and represents the main mechanism of spontaneous bacterial peritonitis. Furthermore, it may sustain the septic process in every type of infection as suggested by the increased levels of endotoxin observed in cirrhotic patients [26]. In addition, innate immune cells display functional abnormalities such as increased production of pro-inflammatory cytokines in response to LPS [27] or alcohol-induced defective phagocytosis [28]. As a consequence, plasma TNF-α and IL-6 levels are higher in cirrhotic patients with bacterial infection than in non-cirrhotic patients [29].

The overall outcome of severe sepsis and septic shock has been improved over the last decade concomitant to the emergence of adjuvant therapeutic interventions such as early-goal directed therapy [30], protective mechanical ventilation with low tidal volumes [13], low-dose corticosteroids [15], intensive insulin therapy [14], or activated protein C [16] supported by positive randomized controlled trials. Although the true benefit of some of these interventions have been addressed by additional studies, they have been included in the Surviving Sepsis Campaign guidelines [8]. This international guideline-based performance improvement program promoted early recognition and management of sepsis and showed that increased compliance with the guidelines was associated with an improved survival rate [31]. In the same way, a continuous decrease in the mortality of severe sepsis and septic shock patients has been reported in large unselected cohorts that mostly comprised non-cirrhotic patients [1,32,33]. Of note, some significant progress in septic shock has also been achieved in highly vulnerable subgroups of patients such as immunocompromised patients with malignancies [9].

Differences in survival between the two periods occurred early in the course of the disorder and were thereafter maintained. The rate of inadequate antibiotic treatment was low and similar between both periods. Most patients received an initial combination antimicrobial treatment of β-lactam associated with either aminoglycosides or fluoroquinolones. An initial single dose of aminoglycosides was commonly subsequently replaced by fluoroquinolones to limit harmful side-effects. Indeed, some retrospective studies suggest a benefit of combination antibiotherapy, most especially of betalactams and aminoglycosides, for septic shock in general cohorts as well as in cirrhotic patients [7,34]. Survival improvement was more likely related to changes in the early management of shock and organ failures. With respect to fluid resuscitation strategies, the majority of patients from the recent period received albumin and consequently received less crystalloids. Albumin resuscitation was associated with higher Child-Pugh and MELD scores (*P *= 0.04 and *P *= 0.03 respectively, data not shown), suggesting that albumin was preferentially indicated to patients with advanced stages of cirrhosis. The role of albumin resuscitation in sepsis remains challenging. A meta-analysis suggested that fluid resuscitation with albumin compared to crystalloids was associated with improved survival in patients with severe sepsis [35]. Specifically, albumin resuscitation has been shown to reduce mortality and renal impairment in cirrhotic patients treated for spontaneous bacterial peritonitis [36]. Accordingly, frequent albumin resuscitation in the recent period of our study was also associated with less frequent recourse to renal replacement therapy. Altogether, these results and ours suggest a possible benefit of albumin resuscitation in cirrhotic patients with severe sepsis, that remains to be prospectively investigated. The frequent use of low-dose corticosteroids was also a hallmark of sepsis management during the second period. Indeed, adrenal dysfunction frequently occurs in patients with septic shock and is associated with hemodynamic instability, renal dysfunction, and increased mortality [37]. However, the use of low-dose corticosteroids in septic shock remains controversial because of discrepant efficacy data and a possible higher risk of nosocomial infections [15]. Of note, this treatment has been specifically addressed in cirrhotic patients with septic shock. In a case-control study, resolution of shock and survival were higher in hydrocortisone-treated cirrhotic patients [38]. However a randomized controlled trial in septic shock cirrhotic patients was stopped for futility at interim analysis because hydrocortisone did not reduce mortality and was associated with an increase in adverse effects such as shock relapse and gastrointestinal bleeding [39]. In addition to sepsis-specific therapies, general measures for ICU patients such as ventilation with low tidal volumes and glucose control with intravenous insulin therapy were also routinely implemented during the second period.

Cirrhotic patients are commonly perceived as poor candidates for ICU admission because of the very high mortality rates associated with organ failures [40,41]. A general improvement in outcome for cirrhotic patients in the ICU has been recently reported regardless of the type of acute complication [42,43]. Several factors may explain this progress, including a better selection of patients on the basis of previous functional status and advances in the management of acute complications such as variceal bleeding [44], hepatorenal syndrome [45], or septic shock as highlighted in the present study. The extent of organ failures clearly represents a major determinant of outcome, and the performance of SOFA score that nearly reached significance in our multivariate model is in accordance with previous studies [6,46]. Most importantly, we also identified the stage of the underlying liver disease assessed by the Child-Pugh score as an independent prognostic factor of septic shock. Until now, the discrimination of the Child-Pugh score calculated at the time of ICU admission had remained inferior to organ failure scores [6,46,47]. As a matter of fact, we computed Child-Pugh score prior to the acute complication in order to reliably assess the stage of liver disease without any interference from new-onset organ failures. This finding carries major practical implications for the decision-making process. Indeed, this could allow a better selection of patients likely to benefit from intensive care, on the basis of underlying disease's status and realistic therapeutic options including liver transplantation. Nevertheless, an accurate individual prognosis prediction of cirrhotic patients is often difficult at the time of ICU admission, but can be markedly refined after a few days [6,46]. In the light of improved outcomes and limited performance of initial prognostic prediction, critically-ill cirrhotic patients with a reasonable long-term prognosis should be offered a broad intensive care access policy with subsequent reappraisal based on the nature of the acute complication and the evolution of organ failures.

Our study has several limitations that we acknowledge. First, the design was retrospective despite most data were prospectively collected through computerized patient data management system. Therefore, we can only report an association between changes in care and patient outcome over the study period. Second, it was carried out in a single center, with a hepatology unit that is closely involved in the decision-making process and in the management of critically ill cirrhotic patients while in the ICU and after discharge. Third, the limited number of patients may limit the external validity of our findings. However, our survival rates in the latter period are similar to those reported by Arabi et al. (hospital survival 24%) [7] and Levesque et al. (ICU survival 36%) [6]. Fourth, indications for ICU admission or end-of-life decisions might have evolved over the study period, and we cannot exclude that patients from the recent period were more carefully selected or referred earlier to the ICU. Nevertheless, the functional status, the stage of the underlying liver disease and the severity scores were similar between both periods. In addition, arterial blood lactate level as an indicator of prolonged systemic hypoperfusion also suggested similar duration and severity of shock before ICU admission. Altogether, these results suggest that improvements in survival were more likely related to changes in care. Fourth, we failed to link the recent improvement in survival with a single therapeutic change, suggesting that it is more likely related to a combination of interventions. Alternatively, some unrecognized or non-collected data might also influence the outcome. For instance, the functional status assessed by the performance status prior to the acute complication is a major prognostic factor in critically-ill cancer patients [48], and might be more accurate than the Knaus scale in this setting. In the same way, the nutritional status might also be of importance in these patients [49].

## Conclusions

This study reports an encouraging improvement in survival in cirrhotic patients with septic shock that needs to be confirmed in a larger multicenter cohort. Implementation of therapeutic advances in sepsis probably accounted for this result. In addition, the stage of the underlying liver disease appears as an important prognostic factor. Delineation of the long-term prognosis of cirrhosis and the related therapeutic options thus appears essential in order to determine the indications for life-sustaining therapies.

## Key messages

• The current survival rate of septic shock in cirrhotic patients remains low but has improved over the recent years.

• Cirrhotic patients could have benefited from recent advances in the management of septic shock.

• The stage of liver disease prior to the acute complication as assessed by the Child-Pugh score appears to be an independent prognostic factor of hospital mortality.

## List of abbreviations

ARDS: Acute Respiratory Distress Syndrome; CI: Confidence Interval; COPD: Chronic Obstructive Pulmonary Disease; ICU: Intensive Care Unit; IL: Interleukin; INR: International Normalized Ratio; MELD: Model for End-stage Liver Disease; OR: Odds Ratio; RRT: Renal Replacement Therapy; SAPS: Simplified Acute Physiology Score; SOFA: Sequential Organ Failure Assessment; TNF: Tumor Necrosis Factor.

## Competing interests

JC is consultant for LFB and received lecture fees. JPM is consultant and member of the scientific board of LFB, and received lecture fees from LFB, Fresenius and Baxter. JC and JPM were the main investigators of an interventional trial on albumin resuscitation in septic shock. FP received lecture fees from LFB. The authors declare that they have no other competing interests relevant to the field of the manuscript.

## Authors' contributions

BS, BC, and FP designed the study. BS, BC, AS, and NM extracted the data. BS and FP performed the statistical analysis. BS, BC, AS, NM, JC, AC, JDC, VM, JPM, and FP contributed to data analysis. BS and FP drafted the manuscript. All authors read and approved the final version of the manuscript.
